# Supplementary material for: Increased Plasma Levels of Adenylate Cyclase 8 and cAMP Are Associated with Obesity and Type 2 Diabetes: Results from a Cross-Sectional Study
Source: Biology (Basel). 2020 Aug 24;9(9):244. doi: 10.3390/biology9090244 (PMC7563501; doi:10.3390/biology9090244)
Supplement: Supplementary file 1 [file biology-09-00244-s001.pdf]

Table S1. Quantitative analysis of ADCY plasma levels in the study subjects according to gender and age classification.

ADCY Isoforms	Total Population					
	Gender		*p-value*	Age		*p-value*
	Male	Female		<46 years	≥46 years	
ADCY1 ng/ml	8.19 ± 1.33 (72)	8.27 ± 1.63 (106)	0.715	8.61 ± 1.58 (61)	8.04 ± 1.45 (117)	**0.020**
ADCY3 ng/ml	8.71 ± 2.70 (53)	8.78 ± 3.58 (75)	0.936	8.39 ± 3.39 (52)	9.00 ± 3.12 (76)	0.412
ADCY8 ng/ml	12.33 ± 1.70 (76)	12.35 ± 1.68 (109)	0.931	12.11 ± 1.46 (65)	12.47 ± 1.79 (120)	0.142

Data are presented as mean ± SD. Differences in the expression level were tested using Mann Whitney U test for ADCY3 and unequal variance t-test for ADCY1 and ADCY8. ADCY, adenylate cyclase. Statistically significant *p*-values are in bold font.

Table S2. Correlation between plasma levels of ADCY isoforms and biochemical variables.

Phenotype	Unadjusted			Adjusted for Age & Gender *		
	ADCY1			ADCY1		
	r	*p-value*	N	r	*p-value*	N
FPG (mmol/L)	−0.10	0.899	175	0.044	0.586	171
Insulin (U/L)	0.242	**0.008**	119	0.287	**0.002**	115
TGL (mmol/L)	0.201	**0.008**	174	0.247	**0.001**	170
BMI (Kg/m^2)	−0.014	0.852	178	0.021	0.781	174
HBA1C (%)	−0.063	0.414	172	−0.007	0.933	168
**Phenotype**	**ADCY3**			**ADCY3#**		
	r	*p-value*	N	r	*p-value*	N
FPG (mmol/L)	0.022	0.803	126	−0.038	0.679	122
Insulin (U/L)	0.062	0.571	85	0.042	0.704	81
TGL (mmol/L)	-0.017	0.853	125	−0.050	0.582	121
BMI (Kg/m^2)	-0.154	0.082	128	−0.183	**0.040**	124
HBA1C (%)	-0.038	0.680	123	−0.106	0.248	119
**Phenotype**	**ADCY8**			**ADCY8**		
	r	*p-value*	N	r	*p-value*	N
FPG (mmol/L)	0.115	0.123	183	0.098	0.189	179
Insulin (U/L)	−0.004	0.963	125	−0.018	0.841	121
TGL (mmol/L)	0.057	0.447	182	0.045	0.551	178
BMI (Kg/m^2)	0.151	**0.040**	185	0.139	0.060	181
HbA1c (%)	0.188	**0.012**	180	0.174	**0.020**	176

Correlation coefficients (r) were calculated using Pearson's correlation test unless otherwise specified. * Indicates partial correlation adjusted for age and gender. # indicates non-parametric partial correlation adjusted for age. ADCY, adenylate cyclase; FPG: Fasting Plasma glucose, BMI, body mass index; HbA1c, glycated hemoglobin; r, correlation coefficient; TGL, triglycerides. Statistically significant *p*-values are in bold font.

Table S3. Correlation between plasma levels of ADCY isoforms and biochemical variables based on diabetes and obesity status.

Category	Phenotype	ADCY1			ADCY3*			ADCY8		
		r	*p*-value	N	r	*p*-value	N	r	*p*-value	N
**Non-T2D**	Age (Years)	−0.301	**0.004**	90	0.258	**0.018**	83	0.070	0.501	96
	FPG (mmol/L)	0.045	0.679	88	0.238	**0.032**	81	−0.023	0.829	94
	Insulin (U/L)	0.207	0.137	53	0.119	0.419	48	−0.023	0.863	57
	TGL (mmol/L)	0.102	0.342	88	0.028	0.807	81	0.091	0.384	94
	BMI (Kg/m^2)	0.044	0.677	90	−0.117	0.293	83	−0.021	0.841	96
	HBA1C (%)	−0.164	0.130	86	0.069	0.543	79	−0.003	0.978	92
**T2D**	Age (Years)	0.068	0.531	88	0.041	0.791	45	−0.090	0.402	89
	FPG (mmol/L)	0.016	0.884	87	0.210	0.166	45	0.029	0.786	89
	Insulin (U/L)	0.326	**0.007**	66	0.060	0.722	37	−0.103	0.404	68
	TGL (mmol/L)	0.326	**0.002**	86	0.098	0.529	44	−0.034	0.756	88
	BMI (Kg/m^2)	−0.078	0.473	88	−0.115	0.453	45	0.294	**0.005**	89
	HBA1C (%)	−0.032	0.771	86	0.253	0.098	44	0.151	0.161	88
**Non-obese**	Age (Years)	−0.243	**0.024**	86	0.157	0.215	64	0.094	0.379	89
	FPG (mmol/L)	−0.057	0.606	83	0.106	0.411	62	0.007	0.947	87
	Insulin (U/L)	0.139	0.281	62	0.133	0.383	45	−0.002	0.988	65
	TGL (mmol/L)	0.160	0.150	83	0.002	0.989	62	0.040	0.715	87
	BMI (Kg/m^2)	0.004	0.974	86	−0.069	0.587	64	0.170	0.111	89
	HBA1C (%)	−0.041	0.719	80	0.132	0.318	59	−0.092	0.406	84
**Obese**	Age (Years)	−0.086	0.413	92	0.106	0.403	64	0.030	0.769	96
	FPG (mmol/L)	0.044	0.677	92	0.056	0.658	64	0.146	0.156	96
	Insulin (U/L)	0.357	**0.006**	57	0.036	0.826	40	−0.041	0.757	60
	TGL (mmol/L)	0.247	**0.018**	91	0.005	0.970	63	0.051	0.624	95
	BMI (Kg/m^2)	0.127	0.228	92	−0.32	**0.010**	64	0.107	0.301	96
	HbA1c (%)	−0.052	0.623	92	−0.083	0.514	64	0.306	**0.002**	96

Correlation coefficients (r) for ADCY1 and ADCY8 were assessed using Pearson's correlation test and ADCY3* using Spearman correlation test. ADCY, adenylate cyclase; FBG, Fasting Blood Glucose; BMI, body mass index; HbA1c, glycated hemoglobin; r, correlation coefficient; TGL, triglycerides. Statistically significant *p*-values are in bold font.

Table S4. Normality test.

ADCY Isoforms	Total Population			Non-Diabetic			Diabetic		
	Statistic	df	*p*-value	Statistic	df	*p*-value	Statistic	df	*p*-value
ADCY1, ng/mL	0.983	120	0.140	0.984	77	0.47	0.965	43	0.210
ADCY3, ng/mL	0.921	120	**<0.001**	0.979	77	0.23	0.969	43	0.301
ADCY8, ng/mL	0.993	120	0.773	0.991	77	0.84	0.994	43	0.997
